# Tet-ON and Tet-OFF regulation in hypervirulent *Klebsiella pneumoniae*

**DOI:** 10.1128/spectrum.00969-26

**Published:** 2026-07-13

**Authors:** Luisa Lutz, Ipek Ekemen, Diana Rasp, Annika Dietz, Lisa Wenzel, Jakob Triebel, Thomas Bertsch, Gundula Schulze-Tanzil, Bernd Neumann, Joerg Steinmann, Ralph Bertram

**Affiliations:** 1 Institute of Laboratory Medicine and Infectious Diseases, Paracelsus Medical Private University, Nuremberg General Hospital 470426 , Nuremberg, Germany; 2 Technische Hochschule Nürnberg Georg Simon Ohm, Faculty of Applied Chemistry 38920, Nuremberg, Germany; 3 Study Program in Human Medicine, Paracelsus Medical University 470426 , Nuremberg, Germany; 4 Institute of Anatomy and Cell Biology, Paracelsus Medical University 470426 , Nuremberg, Germany; Forschungszentrum Jülich GmbH, Juelich, Germany

**Keywords:** tetracycline, inducible expression, gene regulation, hypervirulent *Klebsiella pneumoniae*, pathogenicity, *Galleria mellonella*

## Abstract

*Klebsiella pneumoniae* is an opportunistic gram-negative bacterial pathogen responsible for a variety of infections in hospitals and in the community. Hypervirulent *K. pneumoniae* (hvKp) strains are characterized by their ability to cause liver abscesses and bloodstream infections. This study describes plasmid-based tetracycline-dependent regulation systems for induction or repression of target genes in hvKp. These plasmids encode a wild-type tetracycline repressor (TetR) or a reverse variant (revTetR), and carry *tet*-sensitive promoters, for anhydrotetracycline-dependent Tet-ON or Tet-OFF regulation, respectively. The dynamic range, determined by *gfp* under *tet* control, was up to 19.3-fold with wt-TetR and 5.5-fold with revTetR. As analyzed for up to 48 h, the reporter-gene responses in both systems were controllable in an inducer concentration-dependent manner. When the virulence-associated factor *peg-344* was induced by the Tet-ON system, two different hvKp strains exerted slightly increased pathogenicity in a *Galleria mellonella* infection model. These *tet* vectors can be useful tools for characterizing potential virulence factors and other genes of interest in an important pathogen.

**IMPORTANCE:**

Hypervirulent *Klebsiella pneumoniae* (hvKp) strains are a serious threat in healthcare environments and beyond. Even in healthy individuals, infections often result in liver abscesses, and the bacteria can spread to multiple infection sites. We here describe two kinds of gene expression control in hvKp, dependent on anhydrotetracycline (ATc). The Tet-ON system uses wild-type tetracycline repressor (TetR), and addition of ATc leads to target gene induction. In the Tet-OFF mode, reverse TetR is co-repressed by ATc, resulting in target gene repression. We have quantified both systems by reporter gene assays and also subjected the hypervirulence marker gene *peg-344* to Tet-ON control. When induced, two different hvKp strains exerted slightly increased pathogenicity in a *Galleria mellonella* (greater wax moth) infection model. These systems can be useful tools for characterizing potential virulence factors and other genes of interest in an important pathogen.

## INTRODUCTION

*Klebsiella pneumoniae* is an opportunistic gram-negative bacterial pathogen. Equipped with various antimicrobial resistances, “classic” *K. pneumoniae* (cKp) cause a range of nosocomial infections, including sepsis and pneumonia. Over the last decades, a second pathotype, termed hypervirulent *K. pneumoniae* (hvKp), has emerged globally (1, 2). These hvKp strains are characterized by distinct clinical manifestations, often resulting in liver abscesses, and by their ability to spread via bloodstream to multiple infection sites. In contrast to cKp, hvKp affect healthy, young, and immunocompetent persons who acquire the infection outside the hospital or healthcare environment (1, 3). Whole-genome sequencing studies of hvKp revealed the presence of plasmids carrying potential hypervirulence-mediating genes, whose complex functions and interactions for *in vivo* pathogenicity are not yet fully understood (4). These genes encode capsule regulators and siderophores (among others), which are not typical virulence factors but play a role in host immune modulation (5). Even though these genes may also be present in cKp, genomic analyses verified that their carriage is associated with hvKp pathogenicity, as shown in mice (6, 7). Another important hypervirulence marker encoded on hvKp plasmids is the putative drug/metabolite transporter PEG344 (8). It might contribute to pathogenicity by acting as a regulator for other virulence genes, but its role is not finally clear. When hvKp was grown in human ascites, a pathologic accumulation of fluid within the peritoneal cavity, increased abundance of *peg-344* RNA was reported (8). It has thus been speculated that PEG344 may transport an unidentified growth factor present in this environment.

**Fig 1:**
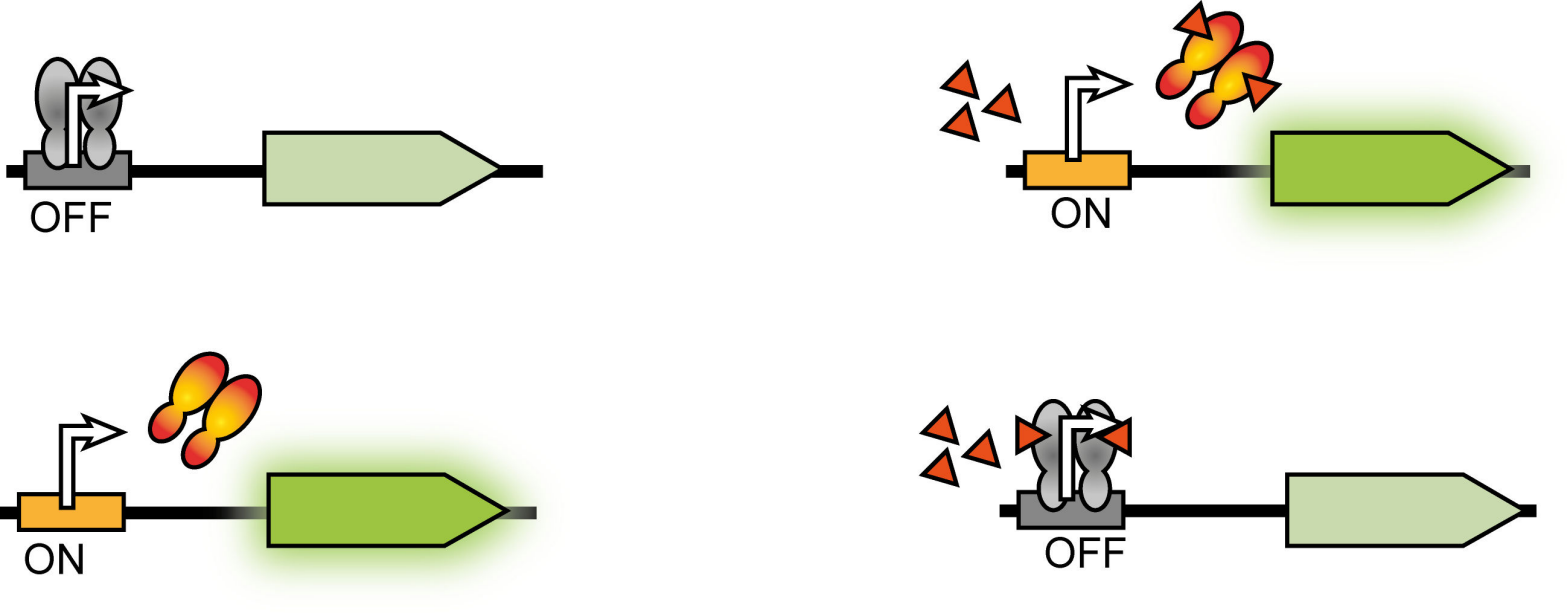
Schematic representation of Tet-ON (above, with wt-TetR) and Tet-OFF regulation (below, with revTetR). In the Tet-ON system, administration of ATc (red triangles) leads to induction, whereas in the Tet-OFF system, ATc administration leads to repression. TetR dimers are symbolized in the DNA-bound (gray) or in the induced/de-repressed state (red-orange). Bent arrows: *tet*-regulated promoters; rectangles: *tetO* (republished from reference (9), previously published under a CC BY-NC-ND 4.0 license).

Besides genome-based studies, the deletion of suspect hypervirulence genes contributes to understanding their role in the pathogenic potential of hvKp. Deletions are an established method in reverse genetics to understand the role of a genetic trait, but are restricted to the binary impact of a gene’s presence or absence. Another possibility to unravel gene-function relationships is to investigate a transcriptional dosage effect by regulated expression. The *tet* system is one of the most versatile and popular tools for gene induction in all kinds of organisms (9–11). To date, *tet* regulation in prokaryotes has reportedly been applied in more than 45 genera across the whole spectrum of bacterial phyla, as well as in archaea. The system originates from tetracycline-resistance determinants, like the one of transposon Tn*10* (12), and is based upon the tetracycline repressor (TetR). To achieve inducible expression, a gene of interest is placed downstream of a *tet*-regulated promoter, which can be of natural or synthetic origin and which carries one or more TetR DNA-binding sites (*tetO*). *Tet* regulation comes in two flavors (Fig. 1). In Tet-ON systems, the gene of interest is repressed by TetR until an inducer, frequently anhydrotetracycline (ATc), is applied. TetR-ATc interaction leads to the detachment of the regulator-effector complex from *tetO* to allow for transcription initiation to occur. Tet-ON is the method of choice if immediate induction from a repressed state is desired. The second mode, Tet-OFF, is most often based upon reverse TetR (revTetR), specific TetR variants with a reversed allostery (13–15). These regulators differ from wt-TetR by one to a few amino acid exchanges only and do not bind to *tetO* unless ATc (here classified as effector) is present. Tet-OFF thus enables a rapid shut-down of a target gene of interest.

We here report plasmid-based Tet-ON and Tet-OFF regulation systems for hvKp. Both types of regulated expression were quantified by reporter gene analysis. Finally, Tet-ON regulation was applied to investigate the effect of *peg-344* induction in two hvKp strains on their pathogenicity in a *Galleria mellonella* (greater wax moth) infection model.

## MATERIALS AND METHODS

### Bacterial strains, growth conditions, phenotypic testing, and manipulations

All cloning was performed in *Escherichia coli* NEB 5-alpha (New England Biolabs [NEB], Frankfurt am Main, Germany). Two clinical isolate *K. pneumoniae* strains were included in this study, SPK0509 (of sequence type [ST] 86) and SPK152528 (of ST29). All bacteria (Table 1) were grown in liquid or on solid lysogeny broth (LB, Carl Roth, Karlsruhe, Germany). Antibiotics were used, where appropriate, at the following final concentrations: ampicillin, 100 µg/mL; chloramphenicol, 60 µg/mL (for selection in *K. pneumoniae*), or 25 µg/mL (for selection in *E. coli*). ATc, the effector of TetR and revTetR, was purchased from Thermo Fisher Scientific (Waltham, MA, USA) and was prepared as a 10 mM stock solution in 70% (wt/vol) ethanol. It was diluted prior to use and was added to bacterial cultures at a final concentration of 0.4 µM unless otherwise stated. Screening for hypervirulence-associated genes was done by using Kleborate (v.2.3.3) (16). Multi-locus sequence typing was performed using the Pathogenwatch online platform (17). Chemically competent *E. coli* was transformed using standard techniques (18). *K. pneumoniae* cells were made electrocompetent as follows: 1 mL of an overnight culture grown in 5 mL of LB with 0.5 µM EDTA (LB/EDTA) was used to inoculate 100 mL of LB/EDTA that was shaken at 37°C until an OD_600_ of 0.7 was reached. After chilling on ice for 20 min, the culture was centrifuged at 4,000 rpm (1,770 × *g*) for 20 min at 4°C. The pellet was resuspended in 15 mL of 10% (vol/vol) ice-cold glycerol and washed twice. Finally, the pellet was resuspended in 1 mL of ice-cold 10% (vol/vol) glycerol. For transformation, 1 µL–2 µL, containing at least 200 ng of plasmid DNA, was added to 50 µL of competent cells in an electroporation cuvette with a 2 mm slot width. Electroporation was performed at 2.5 kV, 25 µF, and 200 Ω. After the pulse, 1 mL of LB was added, and cells were allowed to recover at 30°C with shaking at 300 rpm for 1 h. Cells were plated onto LB plates supplemented with appropriate antibiotics and incubated for up to 2 days at 37°C.

**TABLE 1:** Bacterial strains used in this study

Genus and species	Strain designation	Description/genotype	Reference or source
*Escherichia coli*	NEB 5-alpha	*fhuA2::IS2* Δ(*mmuP-mhpD*)*169* Δ*phoA8 glnX44* ϕ80d[Δ*lacZ58*(*M15*)] *rfbD1 gyrA96 luxS11 recA1 endA1 rph*^WT^ *thiE1 hsdR17*	New England Biolabs (Frankfurt am Main, Germany)
*Klebsiella pneumoniae*	SPK0509	WGS-based typing by Kleborate: ST86, capsule type KL2; hypervirulence-associated genes: aerobactin, salmochelin, *rmpADC*, *rmpA2*, *peg-344*; MGE: pKpVP-1, IncHI1B(pNDM-MAR), *repB*-KLEB; only antibiotic resistance gene: SHV-1	Clinical isolate, abscess material (liver); (19)
*Klebsiella pneumoniae*	SPK152528	WGS-based typing by Kleborate: ST29, capsule type KL54; hypervirulence-associated genes: yersiniabactin; MGE: ICEKp3; only antibiotic resistance gene: SHV-187	Clinical isolate, abscess material (mouth); (this study)

### Isolation and sequencing of nucleic acids

Plasmid DNA was prepared from 5 mL overnight cultures of *E. coli* using the Hi Yield Plasmid Mini Kit (Süd-Laborbedarf, Gauting, Germany), according to the manufacturer’s recommendations. Final plasmid constructs were verified by whole-plasmid sequencing applying Oxford Nanopore Technology (Eurofins, Ebersberg, Germany). *K. pneumoniae* isolates were whole-genome sequenced as part of screening for hvKp in routine diagnostics, as described previously (20, 21).

### Construction of plasmids

All plasmids used in this study are summarized in Table 2. In order to obtain pRAB101-sfgfpΔtetR, plasmid pRAB101-sfgfp (22) was digested with XbaI and self-ligated. This enzyme cuts twice in pRAB101-sfgfp, and the *tetR* gene was almost entirely removed. Plasmid pRAB131-sfgfp was constructed using the Gibson Assembly Cloning Kit from NEB as follows: plasmid pBBR1MCS-1 (23) was linearized with HindIII. The *tetR* gene, the *tet*-regulatory sequence of Tn*10* (P_A_, P_R1_, and P_R2_), and *sfgfp* were amplified from pRAB100 (22) with primers Tn10promotergfp_fwd and Tn10promoter-gfp_rev. A *revtetR* allele termed tetR#28 was amplified from plasmid pTE-28 S15-0X (24) with primers revtetR_fwd and revtetR_rev. Plasmid pRAB131-N25-sfgfp was generated by cloning a synthetic fragment containing the P_N25_ promoter sequence (25) upstream of *revtetR* via SgrAI and AscI. This sequence was ordered from Thermo Fisher Scientific (Waltham, MA, USA). Notably, cloning was successful only after a double-stranded DNA fragment containing another SgrAI site had been added (26). This fragment was obtained by hybridizing oligonucleotides SgrAI_linker_fw and SgrAI_linker_rev. Plasmid pRAB101-peg-344 was constructed by cloning a PCR product generated from *K. pneumoniae* SPK0509 chromosomal DNA (primers: peg-344-fw and peg-344-rev) into pRAB101e via KnpI and SalI. Oligonucleotides used in this study (Table 3) were purchased from Biomers (Ulm, Germany), and restriction enzymes were from NEB (Frankfurt am Main, Germany).

**TABLE 2:** Plasmids used in this study

Designation	Relevant features/characteristics	Reference or source
pBBR1MCS-1	*rep*, *oriV*, *mob*, *cat* (Cm^R^), broad host-range vector	(23)
pTE-28 S15-0X	Source of codon-adapted *revtetR* (tetR#28)	(24)
pRAB100	Source of the Tn*10 tet*-regulatory sequence (P_A_, P_R1_, and P_R2_), *tetR*, and *sfgfp*	(22)
pRAB101e	*tetR*, *tet*-regulatory sequence	(22)
pRAB101-sfgfpΔtetR	pRAB101-sfgfp with *tetR* deleted	This study
pRAB101-sfgfp	pRAB101e with codon-adapted *sfgfp*	(22)
pRAB101-peg-344	pRAB101e with *peg-344*	This study
pRAB131-sfgfp	pRAB101-sfgfp derivative with *tetR* replaced by *revtetR*	This study
pRAB131-N25-sfgfp	pRAB131-sfgfp with additional promoter P_N25_ upstream of *revtetR*	This study

**TABLE 3:** Oligonucleotides used in this study

Designation	Sequence (5′ → 3′)
peg-344-fw	GATCGTCGACTGAAGGGGAATGAATGCGTA
peg-344-rev	GATCGGTACCTCTACACTATATTTTCAGCTATTCA
revtetR_fwd	GCTGCAGGAATTCGATATCAGAGTCATGAGGTTACCATGGTC
revtetR_rev	TTAGGAATTAATGTCCCGCCTGGACAAG
SgrAI_linker_fw	CGCGCCAGGCCGCCACCGGCGGGTCGCCGACGG
SgrAI_linker_rev	CGCGCCGTCGGCGACCCGCCGGTGGCGGCCTGG
Tn10promotergfp_fwd	GGCGGGACATTAATTCCTAATTTTTGTTGACACTC
Tn10promoter-gfp_rev	CGAGGTCGACGGTATCGATATGGAATTGTGAGCGGATAAC

### Quantitative analysis of reporter gene activity

Culture growth and green fluorescence as a means of *tet*-dependent expression of *superfolder-gfp* (*sfgfp*) was quantified as follows. A total of 5 µL of overnight cultures of *K. pneumoniae* SPK0509 was used to inoculate 150 µL of LB in 96-well plate format, supplemented with a final concentration of 60 µg/mL of chloramphenicol (Cm60) and ATc at various concentrations up to 0.8 µM (where appropriate). Multi-mode plate readers used were either a SYNERGY 2 (Agilent Technologies, Winooski, VT, USA) or an Infinite M200 (Tecan, Männedorf, Switzerland). In either device, cultures were grown at 37°C with orbital shaking for 10 s at 200 rpm every 20 min, followed by measurement of cell density (absorbance at 600 nm) and fluorescence (excitation filter: 485 nm with 9 nm bandwidth, emission filter: 520 nm with 20 nm bandwidth). Measurements were conducted for up to 48 h. Each measurement was performed using at least three biological and three technical replicates.

### Confocal laser scanning microscopy (CLSM)

CLSM was applied to qualitatively analyze green fluorescence of reporter strains. Therefore, 100 µL of overnight cultures of *K. pneumoniae* SPK0509 were used to inoculate 5 mL of LB/Cm60 with ATc at various concentrations up to 0.4 µM (where appropriate). Cells were incubated at 37°C in an orbital shaker at 300 rpm for 6 h, avoiding direct exposure to light due to the photosensitivity of ATc (27). One milliliter of the culture was sedimented by centrifugation, washed in 1 mL of phosphate-buffered saline (PBS), resuspended in 20 µL of PBS, and mixed with 20 µL of ProLong Live Antifade Reagent to prevent photobleaching (Thermo Fisher Scientific, Waltham, MA, USA). A total of 10 µL of the suspension were spotted onto a glass slide and covered by a coverslip. CLSM was performed using an SPE-II (Leica Microsystems GmbH, Wetzlar, Germany) with a 63×/1.30 oil objective and Leica X 3D software. The sfGFP protein was excited with 415 nm–477 nm laser light, and fluorescence emission was detected at 488 nm–561 nm.

### *Galleria mellonella* infection model

Larvae of *G. mellonella* (greater wax moth) were purchased from Euro Esche (Quinzano d’Oglio, Italy). The animals used in this study had an average weight of 512 mg (standard deviation: 33 mg) and an average length of 3.2 cm (standard deviation: 0.2 cm) and showed no signs of melanization (blackening) at the onset of the experiments. Overnight cultures of *K. pneumoniae* strains harboring different plasmids were used to inoculate 10 mL of LB/Cm60 and 0.4 µM ATc (where appropriate) to a start OD_600_ of 0.1. After growth for 3 h at 37°C at 300 rpm, the cells were collected and washed in PBS. Then they were resuspended in about 1.5 mL of PBS and adjusted to contain about 2 × 10^6^ cells per mL. The larvae were infected with 5 µL (containing about 1 × 10^3^ cells) of the inoculum through the left proleg using a 100 µL Hamilton Microliter syringe (710N, Hamilton, Reno, NV, USA). The syringe was thoroughly flushed with sterile water, ethanol, and PBS after each injection. Larvae were then incubated in the dark at 37°C in Petri dishes (10 larvae per dish). The bacterial burden of the doses was confirmed by plating cells onto LB/Cm60 agar. Survival of infected larvae was scored at five time points within 72 h. The larvae were considered dead when they failed to respond to touch, which was equivalent to complete melanization. Three to four replicates were performed with different batches of larvae, resulting in 30 to 40 larvae for each strain and condition tested. At the end of each experiment, surviving larvae were terminated by incubation at −20°C. Kaplan-Meier survival curves were plotted using GraphPad Prism 10.4.0, and survival analysis and statistical significance were determined using the log-rank test (GraphPad Software, San Diego, CA, USA).

## RESULTS

### Plasmids for Tet-ON and Tet-OFF regulation in hypervirulent *K. pneumoniae* strains

Our previous study in *Stenotrophomonas maltophilia* (22) had yielded plasmids derived from pBBR1MCS (23) carrying all required components for *tet* regulation. We hypothesized that these might also work in *K. pneumoniae* and selected two clinical isolate hypervirulent strains, SPK0509 and SPK152528, for the following experiments.

To rule out growth impediments by the effector, both strains were cultivated in LB medium with concentrations of up to 1,000 ng/mL (equalling about 2 µM) of ATc and growth was monitored by OD_600_ for 48 h. ATc treatment only exerted small perturbations on growth in either strain (Fig. S1). This suggested that ATc concentrations sufficient for full induction in many other bacteria could also be applied to these hvKp to enable efficient *tet* regulation without impeding growth.

**Fig 2:**
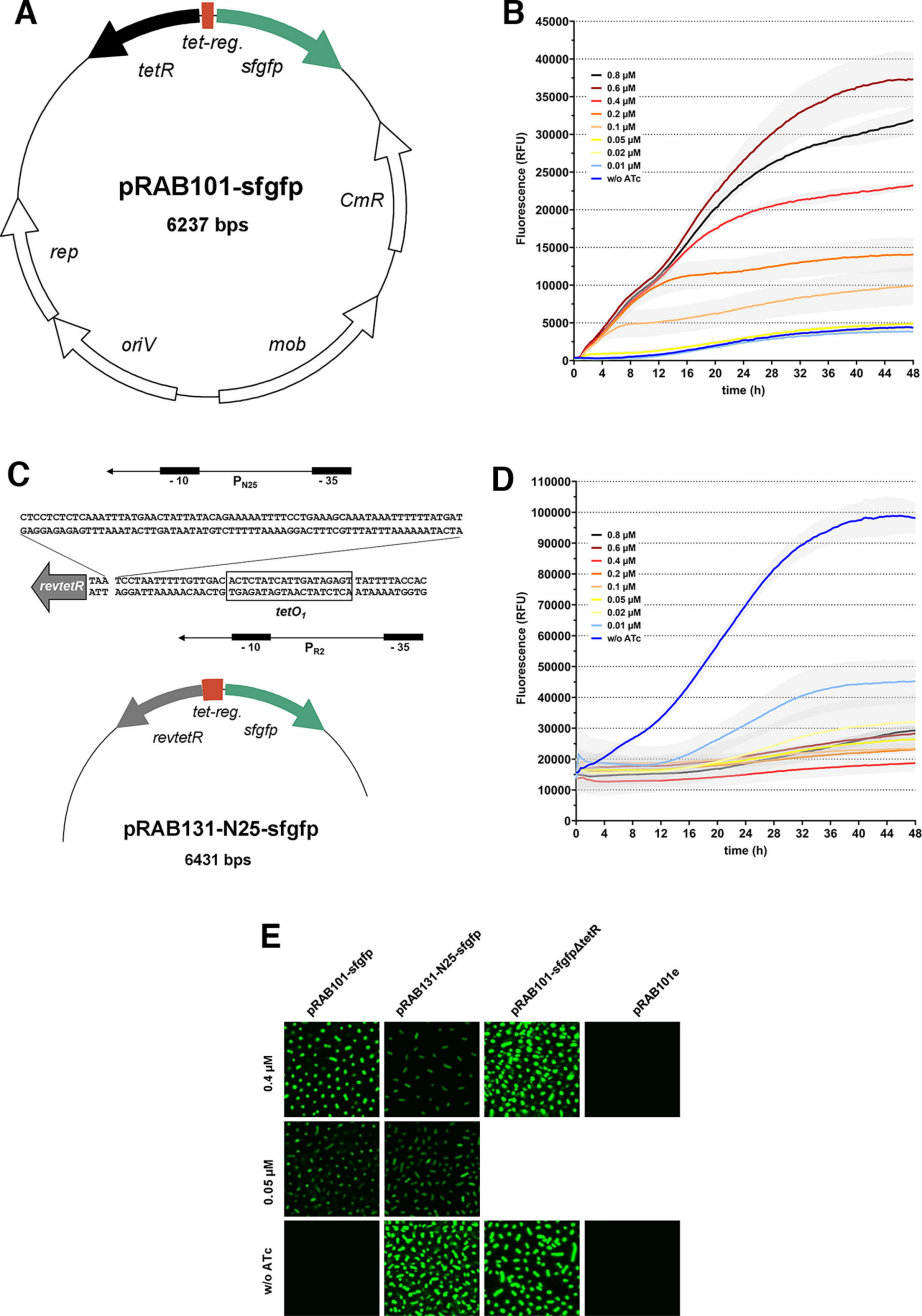
(**A**) Map of plasmid pRAB101-sfgfp. Features are drawn to scale. (**B**) Fluorescence measurements of *K. pneumoniae* SPK0509 (pRAB101-sfgfp) in a time- and inducer concentration-dependent manner. Standard deviations are indicated by gray regions. The inset describes the ATc concentrations used in each experiment. RFU, relative fluorescence units. (**C**) Lower part: partial map with features of plasmid pRAB131-N25-sfgfp. Other features identical to those of pRAB101-sfgfp were omitted. Upper part: sequence of the P_R2_ promoter and the downstream positioned P_N25_ promoter. (**D**) Fluorescence measurements of *K. pneumoniae* SPK0509 (pRAB131-N25-sfgfp). See panel **B** for details. Note the different scale of the *y*-axis. (**E**) CLSM of *K. pneumoniae* SPK0509 carrying plasmids as indicated, grown at ATc concentrations as given to the left.

Two constructs intended for analysis and quantification of *tet* regulation in *K. pneumoniae* had been cloned before: plasmid pRAB101-sfgfp (Fig. 2A), containing an *sfgfp* gene under control of a *tet* regulatory sequence, and pRAB101e, containing the *tet* sequence but no reporter gene (22). We here additionally constructed plasmid pRAB101-sfgfpΔtetR, in which the sequence upstream of the *sfgfp* reporter is devoid of a functional *tetR* gene, to resemble full induction. The three plasmids were each used to transform strain SPK0509. Green fluorescence was quantified as a readout for expression regulation of *sfgfp* in a time- and inducer-dependent manner. Cultures were supplemented with concentrations from 0.01 µM up to 0.8 µM of ATc, and growth and reporter activity were monitored for up to 48 h. Strains harboring pRAB101-sfgfp displayed a directly correlated inducer-dependent response when cultivated with rising concentrations of ATc up to 0.6 µM (Fig. 2B). At 0.4 µM of ATc (a common concentration for bacterial *tet* induction), we observed an induction factor of 16.7, and with 0.6 µM of ATc, the maximal dynamic range during the measurement was 19.3-fold. Fluorescence of SPK0509 (pRAB101e) was very low irrespective of the presence or absence of ATc (Fig. S2A), while SPK0509 (pRAB101-sfgfpΔtetR) produced strong green signals (exceeding those of induced pRAB101-sfgfp) under both conditions (Fig. S2B).

With the aim to also provide Tet-OFF control, we constructed a plasmid similar to pRAB101-sfgfp, but with wt-*tetR* replaced by a *revtetR* variant. The resulting plasmid, pRAB131-sfgfp, failed to achieve ATc-dependent regulation but resembled a fully induced state with or without effector (Fig. S2C). We hypothesized that this might be due to insufficient levels of revTetR. To overcome this limitation, the constitutive P_N25_ promoter stemming from coliphage T5 was cloned between the P_R2_ promoter and the *revtetR* gene of pRAB131-sfgfp to yield pRAB131-N25-sfgfp (Fig. 2C). Indeed, SPK0509 (pRAB131-N25-sfgfp) exhibited drastically decreased green fluorescence in the presence of, but not in the absence of, ATc, indicative of functional Tet-OFF control. As depicted in Fig. 2D and, similarly to the Tet-ON system, regulation was time- and dose-dependent and resulted in a dynamic range peaking at 5.4-fold with 0.4 µM ATc during the course of measurement. No further increase of co-repression was observed at higher effector concentrations.

CLSM was conducted with strains bearing the different *tet*-plasmids. Results depicted in Fig. 2E confirmed findings from quantitative measurements. In the case of SPK0509 (pRAB101-sfgfp), green fluorescence was virtually undetectable in the absence of inducer. At 0.05 µM ATc, cells glowed dimly green, and induction with 0.4 µM of ATc resulted in bright fluorescence. The response appeared homogeneous within the population observed under the microscope, irrespective of the concentration of inducer. Also, SPK0509 (pRAB131-N25-sfgfp) showed uniform and gradually decreased green fluorescence in the presence of 0.05 or 0.4 µM of ATc, respectively, compared to effector-free conditions. Under any condition tested, green fluorescence of SPK0509 (pRAB101e) was below the visible limit in CLSM, whereas SPK0509 (pRAB101-sfgfpΔtetR) showed a strong signal.

### Effects of inducible expression of *peg-344* in hvKp in the *Galleria mellonella* infection model

**Fig 3:**
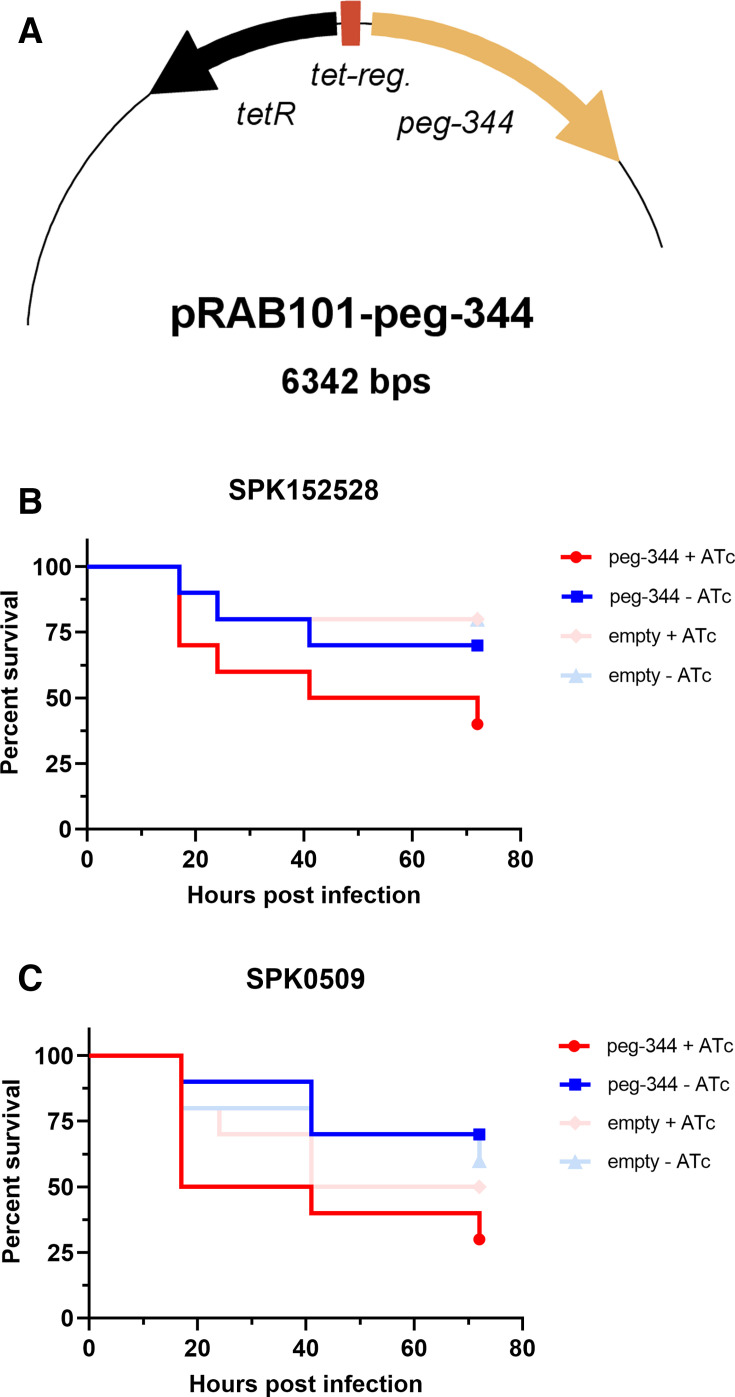
(**A**) Partial map with features of plasmid pRAB101-peg-344. Other features identical to those of pRAB101-sfgfp were omitted. (**B**) Pathogenicity of strains SPK152538 (natively *peg-344*-negative) carrying pRAB101-peg-344 or pRAB101e and cultivated with or without ATc in *G. mellonella* larvae. Survival curves were analyzed using the Kaplan-Meier method (log-rank test). Results obtained with strains carrying pRAB101e (“empty”) are given in faint colors. (**C**) Analogous experiments with SPK0509 (natively *peg-344*-positive) carrying each of the two different plasmids.

In order to place a native *K. pneumoniae* gene under *tet*-inducible control, we chose the virulence-associated factor *peg-344*. The resulting plasmid to achieve Tet-ON control was termed pRAB101-peg-344 (Fig. 3A). Besides strain SPK0509, which is natively *peg-344*-positive, strain SPK152528, which is lacking *peg-344* in the genome, was also transformed with pRAB101-peg-344 or with pRAB101e as a control. The four strains were injected into four separate groups of *G. mellonella* larvae, whose survival was monitored for up to 72 h. Both strains harboring pRAB101-peg-344 imposed an ATc-dependent increased risk of killing the larvae. This effect was at the edge of significance in SPK152528 (Fig. 3B), resembling a *peg-344* complementation scenario (*P* = 0.055), and was also reproducibly recognized in SPK0509 (Fig. 3C), in which *peg-344* was inducibly expressed in addition to the native copy (*P* = 0.279). Larvae that had been infected with hvKp strains bearing pRAB101e had no significant difference in survival, irrespective of absence or presence of ATc, as reflected by *P*-values >0.999 (SPK152528) or 0.627 (SPK0509), respectively.

## DISCUSSION

The major goal of this study was the construction of new and enhanced *tet*-regulation components, originating from *E. coli*, for use in *K. pneumoniae*, particularly hvKp. A comparative analysis of regulatory elements between these two gammaproteobacterial genera confirmed identical sequence motifs in native promoters (28). Thus, induction systems that originate from (or were developed for) the one may generally be suited also for the other (29). Gene-regulation systems previously applied in *K. pneumoniae* make use of a thermosensitive bacteriophage P1-derived C1-regulated promoter (30) or exploit arabinose (31), isopropyl-β-D-thiogalactoside (29), rhamnose, or xylose (32) as inducers. However, the use of thermal induction in higher infection models is limited due to homeostatic temperature conditions, while carbohydrate-based regulation systems may influence metabolism of bacteria and possibly also of the host organism.

The *tet* system is a popular and frequently used gene regulation system and offers unparalleled versatility (9). An early example of ATc-dependent regulation in the genus *Klebsiella* was described in a study examining a nitrogen fixation gene cluster in *K. oxytoca* (33). In *K. pneumoniae*, Wang et al. (34) applied *tet* induction for induced expression of single guide RNA within a system of clustered regularly interspaced palindromic repeats interference. We have previously established *tet* regulation in the gammaproteobacterium *S. maltophilia*. The underlying plasmid pRAB101-sfgfp showed comparable performance also in *K. pneumoniae* (Fig. 2B and reference 22). The present study also marks the implementation of revTetR-based Tet-OFF control in *Klebsiella*. This kind of transcriptional regulation permits target gene repression upon addition of an effector, in contrast to many other induction systems for bacteria, which are suitable to achieve an effector-dependent ON response only. In the present study, both Tet-ON and Tet-OFF could be fine-tuned by adjusting different ATc levels at subinhibitory concentrations (Fig. 2B and D and Fig. S1). One drawback of ATc is its instability upon longer exposure to light, which may result in undesired changes in inducer concentrations, as shown in *E. coli* (27).

The revTetR variant we used is characterized by the mutations EA15 LG17 LV25, which yield an efficient reverse phenotype in different gram-negative and gram-positive bacteria (15, 24, 35–38). The encoding allele chosen in our study had previously been adapted to high G+C codon usage of mycobacteria (24). Since the *K. pneumoniae* genome has a more balanced nucleotide content, the failure of pRAB131-sfgfp to achieve Tet-OFF control in this bacterium might be due to insufficient revTetR protein levels. Supported by examples from literature (24, 36, 38, 39), a strong and constitutive promoter driving *revtetR* was expected to improve Tet-OFF control. Promoter P_N25_ had been used for *tet* regulation in *E. coli* before (25) and also provided improved regulatory effects in our experiments (Fig. 2D).

The Tet-ON system was additionally applied for the induction of a virulence-associated hvKp gene *peg-344* in an *in vivo* infection model. Despite being below the limit of significance, the results of these experiments (Fig. 3B and C) bolster our findings on ATc-dependent induction of the *sfgfp* reporter gene and are in line with previous studies. In a mouse infection model, Bulger et al. found that *peg-344* plays a decisive role in the virulence of pulmonary hvKp infections (8). Russo and colleagues reported an increased risk of severe sickness in mice to be associated with *peg-344* (3). Studies also concluded that strains with a single knockout of *peg-344* do not show a non-pathogenic phenotype (40). Notably, *peg-344* in combination with other virulence genes is strongly associated with the hypervirulence phenotype (3, 7), but not by itself (4). At this point, the actual relevance of *peg-344* cannot finally be assessed.

The validation of *G. mellonella* as an infection model in our laboratory required experimental standardization and a careful choice of suitable larvae, in terms of fitness (as judged by melanization) and larval stage of development. A previous study addressed difficulties of the *G. mellonella* infection model to differentiate between cKp and hvKp (41), whereas the present work included only the latter type of *K. pneumoniae*. Regarding a debate about the general suitability of *G. mellonella* for infection studies with hvKp (41, 42), our study generally suggests that meaningful results can be achieved with this organism, as also stated recently (43). A limitation of our study lies in the fact that we only used two hvKp strains, with quantifications of reporter gene activity assessed in one of them. In fact, genetic backgrounds of clinical isolates compared to laboratory-adapted strains play an important role, also pertaining to transformation efficiency. For example, isolate SPK152528 is neither characterized by a hypermucoid phenotype nor by carriage of various plasmids. Possibly, the integrative conjugative element ICEKp3 or production of yersiniabactin affected the experiments.

### Conclusions

The new vector-based Tet-ON and Tet-OFF systems permit two different types of time- and dose-dependent transcriptional regulation in *K. pneumoniae* and are potentially suitable for investigation of hypervirulence-associated genes in an infection model. Future studies should investigate *tet* regulation also in other cKp or hvKp strains and address the question of whether induction can be achieved even after infection of the larvae (e.g., by force-feeding the animals with an aqueous ATc solution).

Regarding *peg-344* as one of the signature factors for hvKp (44), its exact role in virulence remains elusive. Further studies are warranted, and inducible expression might provide a valuable contribution.

## Data Availability

The data presented in this study, such as plasmid sequences, are available on request.

## SUPPLEMENTAL MATERIAL

Supplemental legends spectrum.00969-26-s0001.docx Descriptive legends for Fig. S1 and S2.

Fig S1A spectrum.00969-26-s0002.tif Growth curve of K. pneumoniae SPK152528 in the presence of ATc at different concentrations.

Fig S1B spectrum.00969-26-s0003.tif Growth curve of K. pneumoniae SPK0509 in the presence of ATc at different concentrations.

Fig S2A spectrum.00969-26-s0004.tif Fluorescence measurement of K. pneumoniae SPK0509 carrying pRAB101e in a time- and inducer concentration-dependent manner.

Fig S2B spectrum.00969-26-s0005.tif Fluorescence measurement of K. pneumoniae SPK0509 carrying pRAB-101-sfgfpΔtetR in a time- and inducer concentration-dependent manner.

Fig S2C spectrum.00969-26-s0006.tif Fluorescence measurement of K. pneumoniae SPK0509 carrying pRAB131-sfgfp in a time- and inducer concentration-dependent manner.

ASM does not own the copyrights to Supplemental Material that may be linked to, or accessed through, an article. The authors have granted ASM a non-exclusive, world-wide license to publish the Supplemental Material files. Please contact the corresponding author directly for reuse.

## References

[1] Hetta HF, Alanazi FE, Ali MAS, Alatawi AD, Aljohani HM, Ahmed R, Alansari NA, Alkhathami FM, Albogmi A, Alharbi BM, Alanzi HS, Alaqyli AB, Ramadan YN. 2025. Hypervirulent *Klebsiella pneumoniae*: insights into virulence, antibiotic resistance, and fight strategies against a superbug. Pharmaceuticals (Basel) 18:724. doi:10.3390/ph18050724 40430542 PMC12115101

[2] Marr CM, Russo TA. 2019. Hypervirulent *Klebsiella pneumonia*e: a new public health threat. Expert Rev Anti Infect Ther 17:71–73. doi:10.1080/14787210.2019.1555470 30501374 PMC6349525

[3] Russo TA, Olson R, Fang C-T, Stoesser N, Miller M, MacDonald U, Hutson A, Barker JH, La Hoz RM, Johnson JR. 2018. Identification of biomarkers for differentiation of hypervirulent *Klebsiella pneumoniae* from classical *K. pneumoniae*. J Clin Microbiol 56:e00776-18. doi:10.1128/JCM.00776-18 29925642 PMC6113484

[4] Stanton TD, Wyres KL. 2024. What defines hypervirulent *Klebsiella pneumoniae*? EBioMedicine 108:105331. doi:10.1016/j.ebiom.2024.105331 39260040 PMC11414540

[5] Russo T.A, MacDonald U, Hassan S, Camanzo E, LeBreton F, Corey B, McGann P. 2021. An assessment of siderophore production, mucoviscosity, and mouse infection models for defining the virulence spectrum of hypervirulent *Klebsiella pneumoniae**.* mSphere 6:e00045-21. doi:10.1128/mSphere.00045-21 33762316 PMC8546679

[6] Russo TA, Alvarado CL, Davies CJ, Drayer ZJ, Carlino-MacDonald U, Hutson A, Luo TL, Martin MJ, Corey BW, Moser KA, Rasheed JK, Halpin AL, McGann PT, Lebreton F. 2023. Differentiation of hypervirulent and classical *Klebsiella pneumoniae* with acquired drug resistance. mBio 15. doi:10.1128/mbio.02867-23 PMC10865842 38231533

[7] Russo TA, Carlino-MacDonald U, Drayer ZJ, Davies CJ, Alvarado CL, Hutson A, Luo TL, Martin MJ, McGann PT, Lebreton F. 2024. Deciphering the relative importance of genetic elements in hypervirulent *Klebsiella pneumoniae* to guide countermeasure development. EBioMedicine 107:105302. doi:10.1016/j.ebiom.2024.105302 39178743 PMC11388194

[8] Bulger J, MacDonald U, Olson R, Beanan J, Russo TA. 2017. Metabolite transporter PEG344 is required for full virulence of hypervirulent *Klebsiella pneumoniae* strain hvKP1 after pulmonary but not subcutaneous challenge. Infect Immun 85:e00093-17. doi:10.1128/IAI.00093-17 28717029 PMC5607406

[9] Bertram R, Neumann B, Schuster CF. 2022. Status quo of tet regulation in bacteria. Microb Biotechnol 15:1101–1119. doi:10.1111/1751-7915.13926 34713957 PMC8966031

[10] Das AT, Tenenbaum L, Berkhout B. 2016. Tet-on systems for doxycycline-inducible gene expression. Curr Gene Ther 16:156–167. doi:10.2174/1566523216666160524144041 27216914 PMC5070417

[11] Sprengel R, Hasan MT. 2007. Tetracycline-controlled genetic switches. Handb Exp Pharmacol 2007:49–72. doi:10.1007/978-3-540-35109-2_3 17203651

[12] Hillen W, Berens C. 1994. Mechanisms underlying expression of Tn10 encoded tetracycline resistance. Annu Rev Microbiol 48:345–369. doi:10.1146/annurev.mi.48.100194.002021 7826010

[13] Kamionka A, Bogdanska-Urbaniak J, Scholz O, Hillen W. 2004. Two mutations in the tetracycline repressor change the inducer anhydrotetracycline to a corepressor. Nucleic Acids Res 32:842–847. doi:10.1093/nar/gkh200 14764926 PMC373327

[14] Resch M, Striegl H, Henssler EM, Sevvana M, Egerer-Sieber C, Schiltz E, Hillen W, Muller YA. 2008. A protein functional leap: how a single mutation reverses the function of the transcription regulator TetR. Nucleic Acids Res 36:4390–4401. doi:10.1093/nar/gkn400 18587152 PMC2490752

[15] Scholz O, Henssler E-M, Bail J, Schubert P, Bogdanska-Urbaniak J, Sopp S, Reich M, Wisshak S, Köstner M, Bertram R, Hillen W. 2004. Activity reversal of Tet repressor caused by single amino acid exchanges. Mol Microbiol 53:777–789. doi:10.1111/j.1365-2958.2004.04159.x 15255892

[16] Lam MMC, Wick RR, Watts SC, Cerdeira LT, Wyres KL, Holt KE. 2021. A genomic surveillance framework and genotyping tool for *Klebsiella pneumoniae* and its related species complex. Nat Commun 12:4188. doi:10.1038/s41467-021-24448-3 34234121 PMC8263825

[17] Argimón S, David S, Underwood A, Abrudan M, Wheeler NE, Kekre M, Abudahab K, Yeats CA, Goater R, Taylor B, Harste H, Muddyman D, Feil EJ, Brisse S, Holt K, Donado-Godoy P, Ravikumar KL, Okeke IN, Carlos C, Aanensen DM, NIHR Global Health Research Unit on Genomic Surveillance of Antimicrobial Resistance. 2021. Rapid genomic characterization and global surveillance of *Klebsiella* using pathogenwatch. Clin Infect Dis 73:S325–S335. doi:10.1093/cid/ciab784 34850838 PMC8634497

[18] Hanahan D. 1983. Studies on transformation of *Escherichia coli* with plasmids. J Mol Biol 166:557–580. doi:10.1016/s0022-2836(83)80284-8 6345791

[19] Aurnhammer F, Baltzer MM, Butt J, Marr LS, Rath A, Kieninger B, Schneider-Brachert W, Utpatel C, Niemann S, Steinmann E, Dechêne A, Steinmann J. 2026. Bernd neumann clinical and genomic profiling of *Klebsiella pneumoniae* in liver abscesses. Virulence 17. doi:10.1080/21505594.2026.2670805 PMC13170388 42118687

[20] Neumann B, Stürhof C, Rath A, Kieninger B, Eger E, Müller JU, von Poblocki A, Gerlitz N, Wollschläger P, Schneider-Brachert W, Schaufler K, Klaper K, Steinmann J. 2023. Detection and characterization of putative hypervirulent *Klebsiella pneumoniae* isolates in microbiological diagnostics. Sci Rep 13:19025. doi:10.1038/s41598-023-46221-w 37923898 PMC10624845

[21] Neumann B, Wilhelm S, Kern JM, Steinmann J. 2025. Cross-border prevalence of putative-hypervirulent *Klebsiella pneumoniae* differs between tertiary hospitals in Austria and Germany. New Microbes New Infect 66:101606. doi:10.1016/j.nmni.2025.101606 40606762 PMC12221275

[22] Horch R, Rasp D, Dietz A, Ebbert R, Steinmann J, Schaible UE, Mamat U, Bertram R. 2023. Tet-dependent gene expression in *Stenotrophomonas maltophilia*. Microbiol Spectr 11:e0157623. doi:10.1128/spectrum.01576-23 37378537 PMC10434252

[23] Kovach ME, Phillips RW, Elzer PH, Roop RM 2nd, Peterson KM. 1994. pBBR1MCS: a broad-host-range cloning vector. BioTechniques 16:800–802. doi:10.1016/0378-1119(81)90080-9 8068328

[24] Klotzsche M, Ehrt S, Schnappinger D. 2009. Improved tetracycline repressors for gene silencing in mycobacteria. Nucleic Acids Res 37:1778–1788. doi:10.1093/nar/gkp015 19174563 PMC2665214

[25] Lutz R, Bujard H. 1997. Independent and tight regulation of transcriptional units in *Escherichia coli* via the LacR/O, the TetR/O and AraC/I1-I2 regulatory elements. Nucleic Acids Res 25:1203–1210. doi:10.1093/nar/25.6.1203 9092630 PMC146584

[26] New England Biolabs. 2026. Restriction Enzyme Cleavage: “single-site” enzymes and “multi-site” enzymes. Available from: https://www.neb.com/en/tools-and-resources/feature-articles/restriction-enzyme-cleavage-single-site-enzymes-and-multi-site-enzymes

[27] Baumschlager A, Rullan M, Khammash M. 2020. Exploiting natural chemical photosensitivity of anhydrotetracycline and tetracycline for dynamic and setpoint chemo-optogenetic control. Nat Commun 11:3834. doi:10.1038/s41467-020-17677-5 32737309 PMC7395757

[28] Kim D, Hong JS-J, Qiu Y, Nagarajan H, Seo J-H, Cho B-K, Tsai S-F, Palsson BØ. 2012. Comparative analysis of regulatory elements between *Escherichia coli* and *Klebsiella pneumoniae* by genome-wide transcription start site profiling. PLoS Genet 8:e1002867. doi:10.1371/journal.pgen.1002867 22912590 PMC3415461

[29] Jiang X, Zhu C, Lin J, Li J, Fu S, Gong H. 2016. Vector promoters used in *Klebsiella pneumoniae**.* Biotechnol Appl Biochem 63:734–739. doi:10.1002/bab.1423 26234465

[30] Schofield DA, Westwater C, Dolan JW, Schmidt MG, Norris JS. 2001. Controlled expression in *Klebsiella pneumoniae* and *Shigella flexneri* using a bacteriophage P1-derived C1-regulated promoter system. J Bacteriol 183:6947–6950. doi:10.1128/JB.183.23.6947-6950.2001 11698385 PMC95537

[31] Iyer R, Moussa SH, Tommasi R, Miller AA. 2019. Role of the *Klebsiella pneumoniae* TolC porin in antibiotic efflux. Res Microbiol 170:112–116. doi:10.1016/j.resmic.2018.11.003 30468763

[32] Shanks R, Ginde R, Stella N, Calvario R, Lehner K, Callaghan J, Komlosi D, Horzempa J. 2025. Generation and comparison of broad-host range inducible expression vectors for use in Gram-negative bacteria including ESKAPE pathogens. Proc W Va Acad Sci 97:58–69. doi:10.55632/pwvas.v97i1.1110 40822576 PMC12352128

[33] Temme K, Zhao D, Voigt CA. 2012. Refactoring the nitrogen fixation gene cluster from *Klebsiella oxytoca**.* Proc Natl Acad Sci USA 109:7085–7090. doi:10.1073/pnas.1120788109 22509035 PMC3345007

[34] Wang J, Zhao P, Li Y, Xu L, Tian P. 2018. Engineering CRISPR interference system in *Klebsiella pneumoniae* for attenuating lactic acid synthesis. Microb Cell Fact 17:56. doi:10.1186/s12934-018-0903-1 29622042 PMC5887262

[35] Debowski AW, Sehnal M, Liao T, Stubbs KA, Marshall BJ, Benghezal M. 2015. Expansion of the tetracycline-dependent regulation toolbox for *Helicobacter pylori**.* Appl Environ Microbiol 81:7969–7980. doi:10.1128/AEM.02191-15 26362986 PMC4651083

[36] Kamionka A, Bertram R, Hillen W. 2005. Tetracycline-dependent conditional gene knockout in *Bacillus subtilis**.* Appl Environ Microbiol 71:728–733. doi:10.1128/AEM.71.2.728-733.2005 15691923 PMC546824

[37] Köstner M, Schmidt B, Bertram R, Hillen W. 2006. Generating tetracycline-inducible auxotrophy in *Escherichia coli* and *Salmonella enterica* serovar typhimurium by using an insertion element and a hyperactive transposase. Appl Environ Microbiol 72:4717–4725. doi:10.1128/AEM.00492-06 16820464 PMC1489314

[38] Stary E, Gaupp R, Lechner S, Leibig M, Tichy E, Kolb M, Bertram R. 2010. New architectures for Tet-on and Tet-off regulation in *Staphylococcus aureus**.* Appl Environ Microbiol 76:680–687. doi:10.1128/AEM.02416-09 19966017 PMC2813013

[39] Guo XV, Monteleone M, Klotzsche M, Kamionka A, Hillen W, Braunstein M, Ehrt S, Schnappinger D. 2007. Silencing *Mycobacterium smegmatis* by using tetracycline repressors. J Bacteriol 189:4614–4623. doi:10.1128/JB.00216-07 17483222 PMC1913471

[40] Tu YC, Lu MC, Chiang MK, Huang SP, Peng HL, Chang HY, Jan MS, Lai YC. 2009. Genetic requirements for *Klebsiella pneumoniae*-induced liver abscess in an oral infection model. Infect Immun 77:2657–2671. doi:10.1128/IAI.01523-08 19433545 PMC2708586

[41] Russo TA, MacDonald U. 2020. The *Galleria mellonella* infection model does not accurately differentiate between hypervirulent and classical *Klebsiella pneumoniae*. mSphere 5:e00850-19. doi:10.1128/mSphere.00850-19 31915230 PMC6952204

[42] Xiao L, Nie Z, Zhuang D, Zhou Y, Zhu W. 2025. Revealing fitness and virulence determinants of hypervirulent *Klebsiella pneumoniae* during infection in *Galleria mellonella* using a transposon library. Front Cell Infect Microbiol 15:1643224. doi:10.3389/fcimb.2025.1643224 40918258 PMC12411516

[43] Schaufler K, Schmidt N, Schwabe M, Heiden SE, Fickenscher H, Woh PY, Tien SB, Velavan TP, Song LH, Neumann B, Idelevich EA, Becker K, Krumbholz A, Kocer K, Boutin S, Nurjadi D, Eger E. 2026. Rethinking virulence screening in *Klebsiella pneumoniae*: a case for a standardised *Galleria mellonella* infection model. The Lancet Microbe:101421. doi:10.1016/j.lanmic.2026.101421 42066787

[44] Tang Y, Du P, Du C, Yang P, Shen N, Russo TA, Liu C. 2025. Genomically defined hypervirulent *Klebsiella pneumoniae* contributed to early-onset increased mortality. Nat Commun 16:2096. doi:10.1038/s41467-025-57379-4 40025046 PMC11873152

