# Supplementary material for: Tet-ON and Tet-OFF regulation in hypervirulent Klebsiella pneumoniae
Source: Microbiol Spectr. 2026 Jul 13;14(8):e00969-26. doi: 10.1128/spectrum.00969-26 (PMC13436325; doi:10.1128/spectrum.00969-26)
Supplement: Supplementary file 1 [file spectrum.00969-26-s0001.docx]

**Supplemental Material**

**Tet-ON and Tet-OFF regulation in hypervirulent *Klebsiella pneumoniae***

Luisa Lutz^1,2^, Ipek Ekemen^1,3^, Diana Rasp^1,3#^, Annika Dietz^1,2^, Lisa Wenzel^1,2^, Jakob Triebel^1^, Thomas Bertsch^1^, Gundula Schulze-Tanzil^4^, Bernd Neumann^1^, Joerg Steinmann^1^ and Ralph Bertram^1^

^1^Institute of Laboratory Medicine and Infectious Diseases, Paracelsus Medical University, Nuremberg General Hospital, Nuremberg, Germany

^2^Technische Hochschule Nürnberg Georg Simon Ohm, Faculty of Applied Chemistry, Nuremberg, Germany

^3^Study Program in Human Medicine, Paracelsus Medical University, Nuremberg, Germany

^4^Institute of Anatomy and Cell Biology, Paracelsus Medical University, Nuremberg, Germany

^#^present address: Klinikum Dritter Orden, Gynaecology and Obstetrics Clinic, Munich, Germany

ORCID: Luisa Lutz: 0009-0009-7438-3781; Jakob Triebel: 0000-0002-1989-8645; Gundula Schulze-Tanzil: 0000-0002-9807-9532; Bernd Neumann: 0000-0002-3580-2105; Joerg Steinmann: 0000-0002-3181-3667; Ralph Bertram: 0000-0003-0654-6381

Contact information of corresponding author:

Klinikum Nürnberg

Institute of Laboratory Medicine and Infectious Diseases

Prof.-Ernst-Nathan-Str. 1

90419 Nuremberg, Germany

telephone: +49 911 398 2520

fax: +49 911 398 3266

e-mail: ralph.bertram@klinikum-nuernberg.de

**Keywords.** tetracycline, inducible expression, gene regulation, hypervirulent *Klebsiella pneumoniae,* pathogenicity, *Galleria mellonella*

**LEGENDS TO SUPPLEMENTARY FIGURES**

**Fig. S1:** Growth curves of *K. pneumoniae* SPK152528 **(A)**, or SPK0509 **(B)** in the presence of ATc at different concentrations (representative examples of three measurements are shown). The inset describes ATc concentrations used in each experiment.

**Fig. S2:** Fluorescence measurements of *K. pneumoniae* SPK0509 carrying pRAB101e **(A)**, pRAB-101-sfgfpΔtetR **(B)**, and pRAB131-sfgfp **(C)** in a time- and inducer concentration-dependent manner. Standard deviations are indicated by grey regions. The inset describes the ATc concentrations used in each experiment. RFU: relative fluorescence units. Note the different scale of the Y-axis in **(A)**.
